# Rare‐Earth Substitution Induced Symmetry Breaking for The First Sc‐Based Nonlinear Optical Chalcogenide with High‐Performance

**DOI:** 10.1002/advs.202411960

**Published:** 2024-12-30

**Authors:** Chen‐Yi Zhao, Chun‐Li Hu, Nian‐Tzu Suen, Xiao‐Hui Li, Hai‐Ping Xu, Wenfeng Zhou, Sheng‐Ping Guo

**Affiliations:** ^1^ School of Chemistry and Chemical Engineering Yangzhou University Yangzhou Jiangsu Province 225000 P. R. China; ^2^ State Key Laboratory of Structural Chemistry Fujian Institute of Research on the Structure of Matter Chinese Academy of Sciences Fujian Province Fuzhou 350002 P. R. China; ^3^ Institute of Experimental Physics Free University Berlin 14195 Berlin Germany; ^4^ Yunnan Key Laboratory of Electromagnetic Materials and Devices National Center for International Research on Photoelectric and Energy Materials School of Materials and Energy Yunnan University Kunming Yunnan Province 650091 P. R. China

**Keywords:** crystal structure, nonlinear optics, rare‐earth, scandium, thiophosphate

## Abstract

Chalcogenides are the most important infrared nonlinear optical (NLO) material candidates, and the exploration of high‐performance ones is attractive and challengeable. Hitherto, there is no NLO scandium (Sc) chalcogenides experimentally studied. Here, new quaternary Sc thiophosphate CsScP_2_S_7_ (CSPS) was synthesized by the facile metal oxide‐boron‐sulfur/reactive flux hybrid solid‐state method. It crystallizes in the monoclinic chiral space group *C*2, and the layered structure is composed by the new ScP_2_S_11_ functional motifs built by ScS_6_ octahedra and P_2_S_7_ dimers, and the structure‐performance analysis reveals that the hyperpolarizability of ScP_2_S_11_ is much greater than the assembled units (ScS_6_ and PS_4_), which makes the first NLO Sc chalcogenide CSPS exhibits strong NLO response (0.8 × AGS) and high laser‐induced damage threshold (LIDT) (4.3 × AGS), and a wide bandgap of 3.10 eV. With the coordination number's reduction of rare‐earth (RE) ion and the rearrangement of P_2_S_7_ dimers, the centrosymmetric structure of CsREP_2_S_7_ family can be broken via substitution with the smallest RE element Sc to form the noncentrosymmetric structure. This work not only discovers a new high‐performance infrared NLO material, but also will inspire researchers to explore more potential NLO Sc chalcogenides.

## Graphical abstract

The first Sc‐based chalcophosphate NLO material CsScP_2_S_7_ featuring the new triangular peak‐like functional motif ScP_2_S_11_ exhibits wide band gap (3.10 eV), strong SHG response (0.8 × AGS), high LIDT (4.3 × AGS), moderate birefringence and phase matching behavior. Comprehensive results show that CsScP_2_S_7_ is a promising IR NLO material with giant potential.

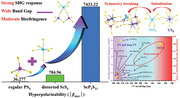

## 1 Introduction

As the important optical component in solid‐state laser device, nonlinear optical (NLO) crystals can convert available laser sources into specific ones by frequency conversion capability.^[^ ^1^ ^]^ Hitherto, there are a variety of commercial NLO crystals in ultraviolet, visible, and near‐infrared regions. Comparatively, the ones in the middle and far‐infrared (MFIR) region including benchmark AgGaS_2_ (AGS), AgGaSe_2_ (AGSe), and ZnGeP_2_ (ZGP) cannot satisfy the market's requirement because of their own deficiencies in low laser‐induced damage threshold (LIDT) or multiphoton absorption,^[^ ^2^ ^]^ which pushes continuous exploration of high‐performance MFIR NLO crystals.^[^ ^3^ ^]^ However, the ones simultaneously possessing large SHG response and high LIDT are still changeable to be obtained.

As the predominant MFIR NLO candidates, chalcogenides have been extensively investigated, including Sn_7_Br_10_S_2_,^[^ ^4^ ^]^ *δ*‐Ga_2_Se_3_,^[^ ^5^ ^]^ Li_4_MgGe_2_S_7_,^[^ ^6^ ^]^ BaHgSe_2_,^[^ ^7^ ^]^ CaMg_6_Ga_6_Se_16_,^[^ ^8^ ^]^ SrZnGeS_4_,^[^ ^9^ ^]^ and Sr_18_Ge_9_O_5_S_31_.^[^ ^10^ ^]^ Surprisingly, though lots of RE chalcogenides like Eu_2_P_2_S_6_,^[^ ^11^ ^]^ LaMg_6_Ga_6_S_16_,^[^ ^12^ ^]^ and La_4_InSbS_9_^[^ ^13^ ^]^ were studied, no Sc‐based ones were studied experimentally. This unusual phenomenon may be attributed to the following reasons. 1) According to the Hard–Soft‐Acid–Base (HSAB) theory, the hard Lewis acid Sc^3+^ ion bonds to the hard O easier than the soft S, resulting in difficulty in synthesis.^[^ ^14^ ^]^ 2) Currently available Sc chalcogenides have band gaps usually < 3.0 eV.^[^ ^15^ ^]^ Their narrow band gaps limit their applications in high‐power devices. Actually, among known Sc‐based compounds, only Sc(IO_3_)_3_ was studied for NLO application.^[^ ^16^ ^]^ Usually, Sc coordinates with Q (Q = S, Se) to form an octahedron, different from the normal high‐coordination configuration for other RE elements. The d^0^ electronic configuration of the Sc^3+^ ion makes the Sc‐centered octahedron distorted under the influence of Jahn–Teller effect, resulting in high hyperpolarizability and large anisotropy.^[^ ^17^ ^]^ More importantly, compared with other d^0^ transition metals (e.g., Ti^4+^, V^5+^) that cause the ultraviolet wavelength to redshift severely, Sc does not significantly weaken the band gap as its 3d empty orbitals are generally in deeper energy levels.^[^ ^18^ ^]^ These features make Sc a special element for the NLO application. Besides, chalcophosphates are receiving increasing interest as the fundamental [PQ_4_]^3−^ unit can be further linked to form a [P_2_Q_*x*_]^4−^ (*x* = 6 or 7) dimer, which makes the structures flexible. Besides, the strong covalency of P–Q bonds is benefit to the SHG effect.^[^ ^19^ ^]^ The combination of Sc with chalcophosphates will maintain the SHG response, and does not weaken the band gap seriously like other heavy elements. Therefore, the LIDT is still possibly high. Combining the above considerations together, Sc chalcophosphates should be a family of potential IR NLO materials. To our knowledge, RE chalcophosphates are very rarely studied as NLO materials,^[^ ^20^ ^]^ and there are no Sc‐based ones studied before.

Recently, we systematically studied NLO RE chalcophosphates.^[^ ^21^ ^]^ Specifically, the CsREP_2_S_7_ family attracted our interest because all known members crystallize with the centrosymmetric (CS) *P*2_1_/*c* structure,^[^ ^22^ ^]^ which may be broken for the smallest Sc‐analogue as its coordination geometry is usually different from the others. Under this consideration, new CsScP_2_S_7_ (CSPS) was obtained by the facile metal oxide‐boron‐sulfur/reactive flux (MOBS/RF) hybrid solid‐state method developed by us after many hard attempts.^[^ ^23^ ^]^ As expected, CSPS crystallizes with the noncentrosymmetric (NCS) *C*2 structure. Here, the crystal chemistry, NLO properties, and corresponding calculations of CSPS were performed. Remarkably, its band gap breaks the 3.0 eV barrier for RE chalcogenides,^[^ ^24^ ^]^ and it exhibits high LIDT (4.3 × AGS), large SHG effect (0.8 × AGS), and moderate birefringence (0.06@2100 nm). Calculation result reveals that novel ScP_2_S_11_ motifs built by ScS_6_ and P_2_S_7_ units contribute to the SHG effect.

## 2 Results and Discussion

**Figure 1:**
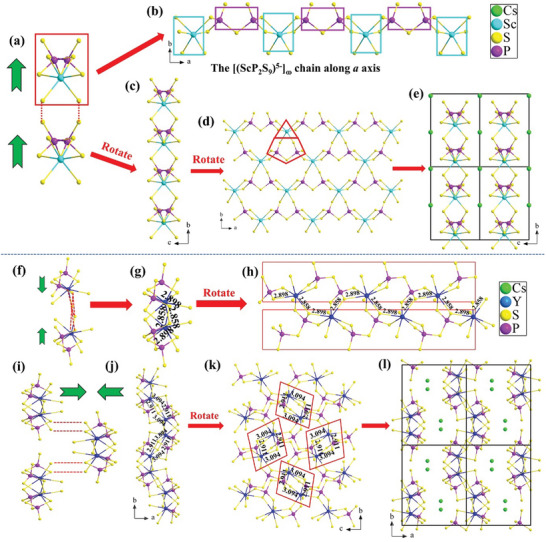
a) [(ScP_2_S_9_)^5–^]_∞_ chain view along *a* direction; b) [(ScP_2_S_9_)^5−^]_∞_ chain viewed along *c* direction; c) {[ScP_2_S_7_]^−^}_∞_ layer viewed along *a* direction; d) {[ScP_2_S_7_]^−^}_∞_ layer parallel to the *ab* plane; e) Crystal structure of CSPS; f) [(YP_2_S_9_)^5−^]_∞_ chain; g) Double stranded [(Y_2_P_4_S_18_)^10−^]_∞_; h) Double stranded [(Y_2_P_4_S_18_)^10−^]_∞_ viewed along *c* direction; i) [(Y_2_P_4_S_18_)^10−^]_∞_ binary chain; j) {[YP_2_S_7_]^−^}_∞_ wrinkle layer viewed along *c* direction; k) {[YP_2_S_7_]^−^}_∞_ wrinkle layer parallel to the *bc* plane; l) Crystal structure of CYPS. The green arrows represent the direction of the dipole moment.

Light yellow plate crystals of CSPS (Figure S1, Supporting Information) were obtained by the MOBS/RF method, and its crystal data are summarized in Tables S1–S3 (Supporting Information). Its PXRD pattern (Figure S2, Supporting Information) proves the sample's purity. CSPS crystallizes in the monoclinic chiral *C*2 space group, and there are one P, one Sc, one Cs, and ten S unique atoms in the asymmetric unit. Every P atom connects with four S atoms to form a distorted PS_4_ tetrahedron, and two neighboring PS_4_ tetrahedra link together to construct a P_2_S_7_ dimer. Each Sc atom coordinates with six S atoms to build a ScS_6_ octahedron, which is further connected with P_2_S_7_ dimers via sharing edges to form [(ScP_2_S_9_)^5−^]_∞_ chains (**Figure** **1**a,b). These chains are connected through sharing S corners to form the {[ScP_2_S_7_]^−^}_∞_ layer along the *ab* plane (Figure 1c,d). The fundamental motif in {[ScP_2_S_7_]^−^}_∞_ layer is ScP_2_S_11_, which is built by one ScS_6_ octahedron and one P_2_S_7_ dimer (Figure 1e). The calculated BVS of Cs, Sc, P, and S are 1.04, 2.95, 5.25, and 2.00–2.30 (Table S4, Supporting Information), respectively, consistent with their ideal oxidation states.

Known CsREP_2_S_7_ (RE = Pr, Nd, Sm–Er, Yb, Y) all crystallize in the CS *P*2_1_/*c* space group, and here CsYP_2_S_7_ (CYPS) is taken as a representative to describe the evolution from CS *P*2_1_/*c* to NCS *C*2 in this family. There is one Cs, one Y, two P, and thirteen S atoms in the asymmetric unit of CYPS. The fundamental units are P_2_S_7_ dimer and YS_8_ bicapped trigonal prism (*btp*), Which are connected to form [(YP_2_S_9_)^5−^]_∞_ chain (Figure 1f). One [(YP_2_S_9_)^5−^]_∞_ chain is connected to the another in the opposite direction via sharing edges to form [(Y_2_P_4_S_18_)^10−^]_∞_ dual‐chain (Figure 1g,h), which are further linked together to form the {[YP_2_S_7_]^−^}_∞_ wrinkle layer along the *ac* plane (Figure 1j–l).

**Figure 2:**
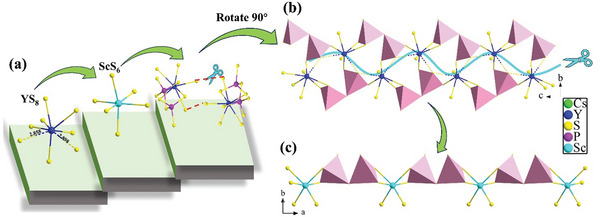
a) YS_8_ bicapped trigonal prism, ScS_6_ octahedron and [(Y_2_P_4_S_18_)^10−^]_∞_ dual‐chain; b) [(Y_2_P_4_S_18_)^10−^]_∞_ dual‐chain viewed along *a* direction; c) [(ScP_2_S_9_)^5−^]_∞_ chain viewed along *c* direction.

By comparing the structures of CSPS and CYPS, they both exhibit distinct layered polyanionic frameworks, featuring [(REP_2_S_9_)^5−^]_∞_ chains connected by P_2_S_7_ dimers. The key difference lies in the increased chain‐to‐chain attraction force in CYPS, attributable to the two important Y─S bonds with the lengths of 2.858(6) and 2.898(9) Å (Figure 2a). These two Y─S bonds stick an additional [(YP_2_S_9_)^5−^]_∞_ chain oriented in the opposite direction, forming a double‐stranded [(Y_2_P_4_S_18_)^10−^]_∞_ (Figure 2b). For CYPS, there is a quadrilateral symmetric center (red parallelogram circled) (Figure 1k). This symmetry is facilitated by larger RE^3+^ ions, which can provide longer bond lengths and form an 8‐coordination environment. However, for smaller Sc^3+^ ions, the absence of longer bond length and the reduction in bond length prevent the formation of this quadrilateral symmetric center. Consequently, the distance between [(REP_2_S_9_)^5−^]_∞_ chains increase and double‐stranded chain breaks into a single chain (Figure 2b,c), leading to structure breaking from CS *P*2_1_/*c* of CYPS to NCS chiral *C*2 structure of CSPS.

This structural transformation inevitably results in a rearrangement of structural units. The attraction between double chains decreases, causing a twist in the direction of interspersed double chains. Similarly, the attraction between single chains diminishes, causing adjacent single chains to twist in opposite directions and align consistently. As a result, the previously wrinkled layer transforms into two simple planar layers composed of [(ScP_2_S_9_)^5−^]_∞_ chains. This alteration likely shifts the dipole moments (Table S5, Supporting Information) from mutual cancellation to summation. Such structural changes suggest that CSPS has a high possibility to possess significant NLO effect. In summary, the radius and coordination number of RE^3+^ ions affect the rearrangement of other atoms, leading to the structure transformation for the AREP_2_S_7_ (A = alkali metal) family.

**Figure 3:**
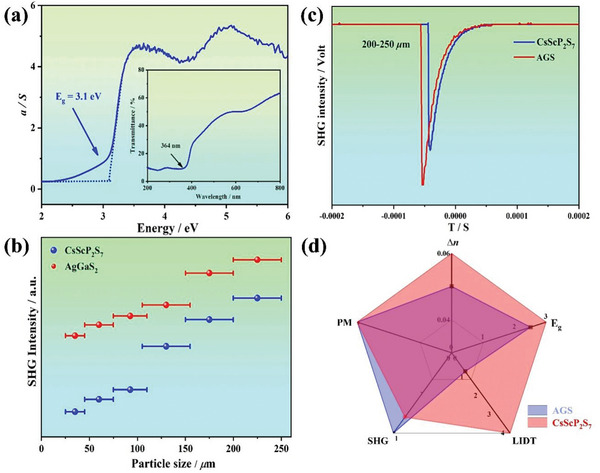
a) Experimental band gap of CSPS; b) SHG intensity of CSPS at 2.1 µm c) SHG signals of CSPS and AGS in the particle size range of 200–250 µm; d) Corresponding radar chart of bandgap E_g_, SHG effect, LIDT, phase‐matching ability and birefringence (∆*n*) for CSPS and AGS.

The band gap (**Figure** **3**a) of CSPS was measured to be 3.10 eV, breaking the 3.0 eV barrier for RE chalcogenides,^[^ ^25^ ^]^ which is much larger than those for benchmark IR NLO crystals like AGS (2.45 eV) and ZGP (1.75 eV). More importantly, CSPS has the shortest ultraviolet cutoff wavelength in the CsREP_2_S_7_ (RE = La, Ce, Pr, Nd, Sm, Gd, Tb, Dy, Ho, Er) family, which can be attributed to the inexistence of *f*–*f* and *d–d* electron transition between RE elements.

As illustrated in Figure 3b, the SHG effect of CSPS gradually increases with increased particle size, suggesting phase matching (PM) behavior. At 200–250 µm, the SHG response reaches to 0.8 × AGS (Figure 3c). As another key factor to evaluate NLO properties, the LIDT of CSPS was evaluated by a single pulse technique.^[^ ^26^ ^]^ The damage threshold for its single crystal is 34.36 MW cm^−2^, around 4.3 × AGS (7.93 MW cm^−2^) (Table S6, Supporting Information). The much enhanced LIDT lays the foundation for its application in high‐power devices. Compared with other reported RE chalcophosphates (Table S7, Supporting Information), the Sc element with the smallest atomic radius and mass in RE family has been neglected for a long time for IR NLO materials application. This work shows that CSPS could be a promising IR NLO material (Figure 3d).

**Figure 4:**
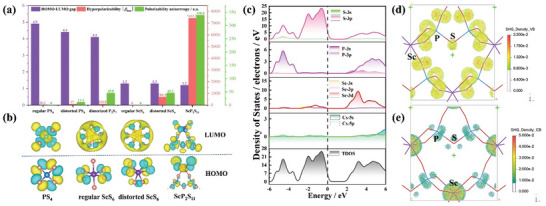
a) The calculated hyperpolarizability, HOMO–LUMO gaps, and polarizability anisortropy of regular [PS_4_], distorted [PS_4_], dimerized [P_2_S_7_], regular [ScS_6_], distorted [ScS_6_] and [ScP_2_S_11_] units; b) The electron cloud of the frontier orbitals of ScS_6_, PS_4_, and ScP_2_S_11_ in LUMO and HOMO; c) Total and partial density of states of CSPS. The Fermi level is set at 0 eV. The SHG density of CSPS in the VB (d) and CB (e).

To better understand the optical properties at the molecular level, the HOMO–LUMO gap and hyperpolarizability (│*β*_max_│) and polarizability anisortropy of regular PS_4_, distorted PS_4_, regular ScS_6_, distorted ScS_6_, and ScP_2_S_11_ units were calculated using the Gaussian program. The hyperpolarizabilities of regular ScS_6_ and PS_4_ units are very small, even close to zero. However, the novel motif ScP_2_S_11_ built by them exhibits a perfect triangular peak‐like geometry with polar *C*_2_ symmetry, and each motif that makes it up undergoes severe structural distortion (**Figure** **4**a). Namely, the dimerization of PS_4_ yielding P_2_S_7_ suffers a symmetry change from *T*_d_ to *C*_2_ *_v_*, leading to a distortion of ScS_6_ toward the polar *C*_2_ direction and a symmetry reduction from *O*_h_ to *C*_2_. The complex motif ScP_2_S_11_ has an extremely high hyperpolarizability, with an overall response much greater than the simple sum of the individual units.

It arises not only from the highly distorted geometry itself but also is closely related to the electronic structure effects. From Figure 4b, it is clear that the HOMOs of the individual ScS_6_ and PS_4_ units are derived from the nonbonding states of S‐3p, and the LUMOs are from the antibonding states of P‐3p/Sc‐3d with S‐3p. They are evenly distributed in each atom. However, the HOMO and LUMO of ScP_2_S_11_ motif show obvious inhomogeneous distribution: the HOMO is mainly in the upper part of ScS_6_, whereas the LUMO is mainly in P_2_S_7_; and there is an obvious variation in the electron cloud of the frontier orbitals for distorted ScS_6_ between the isolated and complex states. It suggests that the electronic polarization of ScP_2_S_11_ in the photoelectric field may be a long‐range process across the small motifs, resulting in extraordinarily large hyperpolarizability. In addition, the introduction of ScS_6_ leads to a smaller HOMO–LUMO gap of ScP_2_S_11_, which also makes the hyperpolarizability enhanced substantially. Most importantly, the polar complex motifs show a perfect parallel arrangement in CSPS, whose space group (*C*2) is in agreement with the symmetry of ScP_2_S_11_ motif (*C*_2_), causing it to produce a large NLO effect.

We also explored the intrinsic source of the strong SHG effect of CSPS from the perspective of electronic structure using density functional theory (DFT) in CASTEP software.^[^ ^27^ ^]^ The calculated band structure shows that CSPS has a direct band gap of 2.01 eV (Figure S3, Supporting Information), which is smaller than the experimental one due to the discontinuity of the exchange‐correlation functional.^[^ ^28^ ^]^ To ensure the accuracy of the calculated band gap of CSPS, the Heyd–Scuseria–Ernzerhof hybrid functional (HSE06) was used to calculate the band gap, and the result of 3.3 eV is very close to the experimental one (Figure S4, Supporting Information). The total and partial density of states (TDOS and PDOS) graphs (Figure 4c) indicate that the upmost valence band (VB) and the bottommost conduction band (CB) are mainly originated from the S‐3p nonbonding states and the unoccupied Sc‐3d orbitals, respectively, suggesting that the band gap of CSPS is dominated by the ScS_6_ units. Also, the overlapping of P‐3p and Sc‐3d with S‐3p electronic states indicates the strong interaction of P─S and Sc─S bonds.

Restricted by the point group 2 and Kleinman symmetry, there are four independent nonzero SHG tensors for CSPS, corresponding to four independent NLO coefficients, namely, *d*_14_ = *d*_25_ = *d*_36_, *d*_16_ = *d*_21_, *d*_22_, *d*_23_ = *d*_34_, among which *d*_36_ gives the largest SHG coefficient of 7.94 pm V^−1^, around 0.65 × AGS (*d*_36_ = 12.5 pm V^−1^) and close to the experimental result. Meanwhile, the calculated birefringence of CSPS is 0.06@2100 nm (Figure S5, Supporting Information), which is sufficient to guarantee PM in the SHG process, consistent with the experimental result. To deeply investigate the contributed orbitals to the SHG effect of CSPS, the SHG‐weighted electron density (SHG density) analysis was performed. As shown in Figure 4d,e, the SHG effect is dominated by the 3p nonbonding states of all the S atoms in VB, and the unoccupied orbitals of Sc‐3d make the main contribution in CB, and P‐3p and S‐3p empty orbitals also give some contributions to SHG. Based on the SHG density, the specific contributions from all the motifs/ions are quantified to be 65.89% for P_2_S_7_ dimers and 34.41% for ScS_6_ octahedra, and the role of Cs^+^ cations is negligible (**Table** **1**). Therefore, it is the ScP_2_S_11_ motif plays a significant role in the large SHG effect of CSPS.

**Table 1:** The SHG‐contributed percentages of the groups/ions in CSPS.

Compound	Group/Ions	SHG Contribute Percentage [%]
CsScP_2_S_7_	P_2_S_7_	65.89
	ScS_6_	34.41
	Cs^+^	−0.41

## 3 Conclusion

In summary, the first NLO‐active Sc chalcogenide CsScP_2_S_7_ is systematically investigated. The structure evolution from CS CYPS to NCS CSPS is well elucidated and gives some important hints for the potential high‐NLO performance of CSPS. The experimental results show that CSPS has a wide band gap, large SHG effect, and high LIDT, indicating its high potential as a new IR NLO material. The HOMO–LUMO gap and hyperpolarizability calculation results suggest that the polar functional motif ScP_2_S_11_ assembled from distorted octahedron (ScS_6_) and dimer (P_2_S_7_) contributes to the wonderful NLO properties of CSPS. The encouraging NLO properties and the scarcity of NLO Sc chalcogenides will definitely attract much interest in this highly undeveloped system.

## 4 Experimental Section

### Synthesis

CSPS crystals were synthesized by the metal oxide‐boron‐sulfur/reactive flux method established by us. The raw materials were CsI (Aladdin, 99.9%), Sc_2_O_3_ (Aladdin, 99.99%), P_2_S_5_ (Macklin, 99%), S (Sinopharm, 99.5%), and B (Aladdin, 99%) with the molar ratios of 4:2:1.35:3:4, which was adopted after many attempts to steadily synthesize CSPS with high yield and good crystal quality. Where CsI was used both as Cs source and flux, Sc_2_O_3_ was used as the Sc source, and B acts as the reducing reagent to capture O from Sc_2_O_3_. By this routine, it was unnecessary to introduce active/expensive Cs or its binary sulfide as the Cs source, and expensive Sc or Ss_2_S_3_ as the Sc source. Using this hybrid solid‐state method, more than one hundred multinary alkali metal sulfides were successfully synthesized by us.^[^ ^29^ ^]^ The mixture was weighed and grown to be a fine powder, which was then pressed into one pellet and loaded into a fused quartz tube. The tube was evacuated to 1 × 10^−4^ Torr and flame‐sealed. Subsequently, the tube was put into a muffle furnace and heated to 1173 K slowly, and kept at this temperature for three days. Finally, the reaction was cooled to room temperature at the rate of 2 °C h^−1^. The product was washed with absolute ethyl alcohol, and the transparent crystals stable in the air were obtained with a yield around 80%.

### Single‐Crystal X‐Ray Diffraction (SXRD) Characterization

The single crystal data of CSPS was collected by Bruker D8 QUEST X ray diffractometer with Mo‐K*α* radiation (*λ* = 0.71073 Å). The crystal structure was solved by Direct Method, and refined by full‐matrix least‐squares techniques on F^2^ with aeolotropic thermal parameters for all atoms using SHELXL‐2019 within the Olex2 interface.^[^ ^30^ ^]^ The crystal data and structure refinement parameters are summarized in Table S2 (Supporting Information), important bond lengths, atomic coordinates, equivalent isotropic displacement parameters, and the bond valence sum (BVS) are shown in Tables S3–S5 (Supporting Information), respectively. The cif document was also deposited with the CCDC number of 2363854.

### Power X‐Ray Diffraction (PXRD) Characterization

The flat surface and the bright color plate crystals were picked out under a microscope and ground evenly in an agate mortar. The power X‐Ray diffraction analysis for the power sample was carried out through Bruker D8 Advance diffractometer (40 kV, 100 mA for Cu‐K*α* radiation, *λ* = 1.5406 Å) with the scan speed of 5° min^−1^ in the range of 5°–70° at room temperature. Experimental PXRD pattern of CSPS matches well with the simulated one generated from the single crystal data by using the Mercury software (Figure S1, Supporting Information), suggesting that the picked out sample was pure.

### UV–Vis–NIR Diffuse Reflectance Spectroscopy

The UV–vis–NIR spectrum of CSPS was obtained through Varian Cary 5000 with a scope of 200–800 cm^‒1^. BaSO_4_ was used as the background, and the Kubelka−Munk formula was applied to convert the reflection spectrum to the absorption spectrum.^[^ ^31^ ^]^

### Power SHG and Single Crystal LIDT Measurements

The powder sample's SHG response of CSPS was measured through the Kurtz–Perry method^[^ ^32^ ^]^ at 2100 nm. The sample was sieved into six distinct sizes of 25–45, 45–75, 75–105, 105–150, 150–200, and 200–250 µm, respectively. The SHG signal of the samples was captured on the oscilloscope, and the same particle size range AGS samples were used as the standards.

A single crystal of CSPS with a flat surface was selected to evaluate the LIDT value, which was radiated by the 1064 nm laser with a pulse width *τ*_p_ of 10 ns. The laser energy output was increased gradually until occurred visible damage on the surface of the sample under the microscope. The similar‐size single crystal of AgGaS_2_ was used as the reference.

### Theoretical Calculations

To further understand the structure‐performance relationship, the first‐principles density functional theory calculations of CSPS were performed by using CASTEP module in Material Studio software.^[^ ^27^ ^]^ The calculations included band structure, density of state (DOS), and optical properties. The model structure was established according to the single crystal structure data and no further geometry optimization. Generalized gradient approximation (GGA) with Perdew–Burke–Emzerh (PBE) functional was adopted for the exchange and correlation function.^[^ ^33^ ^]^ The cut‐off‐energy was 320 eV and the threshold of 5 × 10^‒7^ eV was set for the self‐consistent‐field convergence of the total electronic energy. The numerical integration of the Brillouin zones was performed using 3 × 3 × 4 as Monkhorst–Pack *k*‐point mesh and the Fermi level at 0 eV was selected as the reference. The valence electrons were Cs–5s5p, Sc–3s3p3d, P–3s3p, and S–3s3p.

The static second‐order nonlinear coefficient *χ*_αβγ_^(2)^ was divided into contributions from Virtual‐Hole (VH) and Virtual‐Electron (VE):^[^ ^34^ ^]^

(1) $${\chi_{\alpha \beta \gamma }}^{\left(2\right)}={\chi_{\alpha \beta \gamma }}^{\left(2\right)}\left(\mathrm{VE}\right)+{\chi_{\alpha \beta \gamma }}^{\left(2\right)}\left(\mathrm{VH}\right)$$

*χ*_αβγ_^(2)^ (VE) and *χ*_αβγ_^(2)^ (VH) can be computed as the following formulae:

(2)

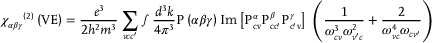



(3)

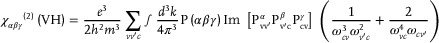




The contribution of the orbitals or bands to the second‐order susceptibility was evaluated by the SHG‐density method. The static second‐order susceptibilities *χ*_αβγ_ can be simplified as:

(4) $${\chi_{\alpha \beta \gamma }}^{\left(2\right)\;}={\chi_{\alpha \beta \gamma }}^{\left(2\right)}\left(\mathrm{VE}\right)+{\chi_{\alpha \beta \gamma }}^{\left(2\right)}\left(\mathrm{VH}\right)+{\chi_{\alpha \beta \gamma }}^{\left(2\right)}\left(\mathrm{two}-\mathrm{bands}\right)$$

Based on sum‐over‐states formalism,^[^ ^35^ ^]^ the SHG coefficient can be calculated. The SHG‐weight electron density can be obtained through summing all SHG‐weight orbitals in VB/CS over different k‐points in space. And the contribution of NLO properties in element orbitals can be observed directly.

The calculations of HOMO–LUMO gap, hyperpolarizability, and polarizability anisotropy were performed by the Gaussian 16 package.^[^ ^36^ ^]^ DFT method at the B3LYP level with 6–31G basis sets was used to calculate the contribution units.

[CCDC 2 363 854 contains the supplementary crystallographic data for this paper. These data can be obtained free of charge from The Cambridge Crystallographic Data Centre via www.ccdc.cam.ac.uk/data_request/cif.]

## Conflict of Interest

The authors declare no conflict of interest.

## Supporting information



Supporting Information

## Data Availability

The data that support the findings of this study are available in the supplementary material of this article.
