# Supplementary material for: Rare‐Earth Substitution Induced Symmetry Breaking for The First Sc‐Based Nonlinear Optical Chalcogenide with High‐Performance
Source: Adv Sci (Weinh). 2024 Dec 30;12(8):2411960. doi: 10.1002/advs.202411960 (PMC11848564; doi:10.1002/advs.202411960)
Supplement: Supplementary file 1 — Supporting Information [file ADVS-12-2411960-s001.docx]

**Supporting Information**

**Rare-Earth Substitution Induced Symmetry Breaking for The First Sc-Based Nonlinear Optical Chalcogenide with High-Performance**

*Chen-Yi Zhao^#^,* *Chun-Li Hu^#^, Nian-Tzu Suen, Xiao-Hui Li, Hai-Ping Xu, Wenfeng Zhou and Sheng-Ping Guo**

**Table of contents**

| **Section** | **Title** |
| --- | --- |
| Table S1 | Crystal data and structure refinement parameters for CsScP_2_S_7_. |
| Table S2 | Important bond lengths (Å) for CsScP_2_S_7_. |
| Table S3 | Atomic coordinates ( × 10^4^) and equivalent isotropic displacement parameters (*U*_eq_^a^, Å^2^ × 10^3^) for CsScP_2_S_7_. |
| Table S4 | Bond valence sum (BVS) in CsScP_2_S_7_. |
| Table S5 | Dipole moments of CsScP_2_S_7_. |
| Table S6 | Laser-induced damage threshold of CsScP_2_S_7_ and AgGaS_2_ for their crystal samples. |
| Table S7 | Reported NLO-active rare-earth chalcophosphates. |
| Table S8 | Known rare-earth chalcophosphates containing PS_4_ unit. |
| Figure S1 | The crystal photograph of CsScP_2_S_7_. |
| Figure S2 | The power X-ray diffraction pattern of CsScP_2_S_7_. |
| Figure S3 | Band structure of CSPS, and the Fermi level is set at 0 eV. |
| Figure S4 | The calculated band structure of CsScP_2_S_7_ using HSE06. |
| Figure S5 | Calculated frequency-dependent birefringence of CsScP_2_S_7_. |
| Figure S6 | Calculated refractive indices of the fundamental and second harmonic wavelengths of CsScP_2_S_7_. |

**Table S1.** Crystal data and structure refinement parameters for CsScP_2_S_7_.

| **Empirical formula** | **CsScP_2_S_7_** |
| --- | --- |
| Formula weight | 464.23 |
| *T*/K | 296 |
| Crystal system | Monoclinic |
| Space group | *C*2 |
| *a*/Å | 8.7492(10) |
| *b*/Å | 9.8135(11) |
| *c*/Å | 6.6902(7) |
| *β/°* | 98.559(3) |
| Volume/ Å | 568.03(11) |
| *Z* | 2 |
| *ρ*_calc_ g/cm^3^ | 2.714 |
| *μ*/mm^–1^ | 5.311 |
| *F* (000) | 436.0 |
| 2*θ* range/° | 6.158 to 55.164 |
| Measd. reflns | 4043 |
| Indep. reflns/R_int_ | 1314/0.0282 |
| GOF on *F*^2^ | 1.064 |
| R1, wR2 (*I* > 2σ(*I*))^a^ | 0.0238, 0.0463 |
| R1, wR2 (all data)^a^ | 0.0273, 0.0474 |
| Δ*ρ*_max_/Δ*ρ*_min_, e/Å^3^ | 1.31/-1.20 |
| Flack parameter | 0.05(2) |

**Table S2.** Important bond lengths (Å) for CsScP_2_S_7_.

| Bond | Distance/ Å | Bond | Distance/ Å |
| --- | --- | --- | --- |
| Cs(1)–S(1)^1^ | 3.7617(14) | Cs(1)–S(4)^1^ | 4.0605(15) |
| Cs(1)–S(1) | 3.7617(14) | Sc(1)–S(1) | 2.5951(17) |
| Cs(1)–S(2)^2^ | 3.5511(8) | Sc(1)–S(1)^3^ | 2.5951(17) |
| Cs(1)–S(2)^3^ | 3.5511(8) | Sc(1)–S(3)^8^ | 2.5910(17) |
| Cs(1)–S(2) | 3.5511(8) | Sc(1)–S(3)^9^ | 2.5910(17) |
| Cs(1)–S(3)^4^ | 3.5503(15) | Sc(1)–S(4)^10^ | 2.5634(13) |
| Cs(1)–S(3)^5^ | 3.5503(15) | Sc(1)–S(4)^1^ | 2.5634(13) |
| Cs(1)–S(4)^6^ | 3.6984(14) | P(1) –S(2) | 2.1399(18) |
| Cs(1)–S(4) | 4.0605(15) | P(1) –S(3) | 2.0140(19) |
| Cs(1)–S(4)^7^ | 3.6984(14) |  |  |

Symmetry transformation used to generate equivalent atoms: ^1^-*x*, +*y*, -*z*; ^2^+*x*, +*y*, 1+*z*; ^3^-*x*, +*y*, -1-*z*; ^4^-1/2+*x*, -1/2+*y*, +*z*; ^5^1/2-*x*, -1/2+*y*, -*z*; ^6^1/2-*x*, 1/2+*y*, -*z*; ^7^ -1/2+*x*, 1/2+*y*, +*z*; ^8^+*x*, -1+*y*, +*z*; ^9^-*x*, -1+*y*, -1-*z*; ^10^+*x*, +*y*, -1+*z*; ^11^1/2-*x*, -1/2+*y*, -1-*z*.

**Table S3.** Atomic coordinates ( × 10^4^) and equivalent isotropic displacement parameters (*U*_eq_^a^, Å^2^ × 10^3^) for CsScP_2_S_7_.

| **Atom** | **Wyckoff site** | ***x*** | ***y*** | ***z*** | ***U*_eq_^a^*/*Å^2^** |
| --- | --- | --- | --- | --- | --- |
| Cs(1) | 2*a* | 0 | 9736.9(7) | 0 | 41.1(2) |
| Sc(1) | 2*b* | 0 | 5778.6(14) | -5000 | 18.4(3) |
| S(1) | 4*c* | 2031.9(17) | 7537.8(14) | -3387(2) | 25.0(3) |
| S(2) | 2*b* | 0 | 10952(2) | -5000 | 18.9(4) |
| S(3) | 4*c* | 1297.7(17) | 13883.6(15) | -2637(2) | 26.9(3) |
| S(4) | 4*c* | 1653.5(14) | 6075.9(14) | 2172.5(19) | 21.1(3) |
| P(1) | 4*c* | 1946.7(16) | 12256.4(13) | -4142(2) | 15.7(3) |

*^a^U*_eq_ is defined as one third of the trace of the orthogonalized *U_ij_* tensor.

**Table S4.** Bond valence sum (BVS) in CsScP_2_S_7_.

| **Atom** | **BVS** | **Atom** | **BVS** |
| --- | --- | --- | --- |
| **Cs(1)** | 1.043258947 | **S(2)** | 2.30226149 |
| **Sc(1)** | 2.954851339 | **S(3)** | 2.044663231 |
| **P(1)** | 5.257831246 | **S(4)** | 2.060241235 |
| **S(1)** | 2.000851177 |  |  |
| GII = 0.15 | | | |

**Table S5.** Dipole moments of CsScP_2_S_7_.

| **CsScP_2_S_7_** | | | | |
| --- | --- | --- | --- | --- |
| Species | Dipole moment/(Debye) | | | |
|  | *x* | *y* | *z* | Total magnitude |
| Sc(1)S_­6_ | -0.006 | -2.992 | -0.000 | 2.992 |
| Sc(2)S_­6_ | -2.828 | -0.315 | -0.712 | 2.933 |
| Sc(3)S_­6_ | 2.828 | -0.315 | 0.712 | 2.933 |
| P(1)S_4_ | 6.702 | -1.113 | 3.632 | 7.703 |
| P(2)S_4_ | -6.700 | -1.113 | -3.639 | 7.706 |
| P(3)S_4_ | 0.731 | 4.768 | -1.029 | 4.933 |
| P(4)S_4_ | -0.729 | 4.768 | 1.032 | 4.933 |
| Unit cell | -0.003 | 3.686 | -0.005 | 3.686 |

**Table S6.** Laser-induced damage thresholds of CsScP_2_S_7_ and AgGaS_2_ for their crystal samples.

| **Compound** | **Damage energy**  **(mJ)** | **Spot area**  **(cm^2^)** | **Damage threshold**  **(MW/cm^2^)** | **Relative value** |
| --- | --- | --- | --- | --- |
| CsScP_2_S_7_ | 7.8 | 0.022698007 | 34.36425069 | 4.3 |
| AGS | 1.8 | 0.022698007 | 7.930211697 | 1 |

**Table S7.** Reported NLO-active rare-earth chalcophosphates.

| Compound | Space group | E_g_ (eV) | SHG  (×AGS) | LIDT  (×AGS) | Ref. |
| --- | --- | --- | --- | --- | --- |
| Eu_2_P_2_S_6_ | *Pn* | 2.54 | 0.9 | 3.4 | [1] |
| K_3_HoP_2_S_8_ | *P*2_1_ | – | 1.1 | 3.0 | [2] |
| K_3_ErP_2_S_8_ | *P*2_1_ | – | 1.2 | 2.5 | [2] |
| K_3_YP_2_S_8_ | *P*2_1_ | 3.37 | 1.4 | 7.0 | [2] |
| KSmP_2_S_6_ | *P*2_1_ | 2.58 | 0.3 | 20.12 | [3] |
| KGdP_2_S_6_ | *P*2_1_ | 3.61 | 0.52 | 16.36 | [3] |
| KTbP_2_S_6_ | *P*2_1_ | 3.65 | 0.35 | 14.79 | [3] |
| KDyP_2_S_6_ | *P*2_1_ | 3.79 | 0.28 | 13.99 | [3] |
| KSmP_2_Se_6_ | *P*2_1_ | 1.92 | 1.08 | 1.43 | [4] |
| KGdP_2_Se_6_ | *P*2_1_ | 2.53 | 0.44 | 4.33 | [4] |
| KTbP_2_Se_6_ | *P*2_1_ | 2.46 | 0.34 | 2.29 | [4] |
| CsScP_2_S_7_ | *C*2 | 3.10 | 0.8 | 4.3 | This work |

**Table S8.** Known rare-earth chalcophosphates containing PS_4_ unit.

| Compound | Space Group | Rare-earth  Units | Chaoclphosphates  Units | Ref. |
| --- | --- | --- | --- | --- |
| Cs_3_Sm[PS_4_]_2_ | *P*2_1_/*n* | [SmS_8_] | [PS_4_] | [5] |
| Rb_3_Sm[PS_4_]_2_ | *P*2_1_ | [SmS_8_] | [PS_4_] | [5] |
| Cs_2_NaLn(PS_4_)_2_  (Ln=La-Nd,Sm,Gd-Ho) | *P*2_1_/*c* | [LnS_8_](Ln=La-Sm)  [LnS_9_](Ln=Gd-Ho) | [PS_4_] | [6] |
| K_3_CeP_2_S_8_ | *P*2_1_/*c* | [CeS_6_] | [PS_4_] | [7] |
| K_3_LaP_2_S_8_ | *P*2_1_/*c* | [LaS_8_] | [PS_4_] | [7] |
| K_4_EuP_2_S_8_ | *Ibam* | [EuS_8_] | [PS_4_] | [8] |
| KEuPSe_4_ | *Pnma* | [EuS_8_] | [PS_4_] | [9] |
| K_9_Nd[PS_4_]_4_ | *C*2/*c* | [NdS_8_] | [PS_4_] | [10] |
| K_3_Nd[PS_4_]_2_ | *P*2_1_/*c* | [NdS_8_] | [PS_4_] | [10] |
| Cs_3_Nd[PS_4_]_2_ | *P*2_1_/*n* | [NdS_8_] | [PS_4_] | [11] |
| K_3_Nd_3_[PS_4_]_4_ | *C*2/*c* | [NdS_8_] | [PS_4_] | [11] |
| Li_4_Ln[PS_4_]Cl  (Ln=Pr,Nd,Sm) | *C*2/*c* | [LnS_8_]  (Ln=Pr,Nd,Sm) | [PS_4_] | [12] |
| Rb_3_Ln[PS_4_]_2_  Ln=La,Ce,Pr | *P*2_1_ | [LnS_8_]  (Ln=La,Ce,Pr) | [PS_4_] | [13] |
| Li_3_La[PS_4_]_2_ | *Pbca* | [LaS_8_] | [PS_4_] | [14] |
| Cs_3_Pr_5_[PS_4_]_6_ | *C*2/*c* | [PrS_8_] | [PS_4_] | [15] |
| Ag_3_RE[PS_4_]_2_  (RE=Y,Dy,Ho,Er) | *C*2/*c* | [RES_8_] | [PS_4_] | [16] |
| K_3_Gd_3_[PS_4_]_4_ | *C*2/*c* | [GdS_8_] | [PS_4_] | [10] |
| K_9_Gd[PS_4_]_4_ | *C*2 | [GdS_8_] | [PS_4_] | [10] |
| K_6_Yb_3_(PS_4_)_5_ | *C*2/*c* | [YbS_8_] | [PS_4_] | [17] |
| Li_9_Nd_2_[PS_4_]_5_ | *C*2/*c* | [NdS_8_] | [PS_4_] | [18] |
| Li_9_Ho_2_[PS_4_]_5_ | *C*2/c | [HoS_8_] | [PS_4_] | [19] |
| Li_9_Yb_2_[PS_4_]_5_ | *C*2/*c* | [YbS_8_] | [PS_4_] | [20] |
| Li_6_Yb_3_[PS_4_]_5_ | *C*2/*c* | [YbS_8_] | [PS_4_] | [21] |
| K_2_NdP_2_S_7_ | *P*2_1_/*n* | [NdS_8_] | [PS_4_][P_2_S_6_] | [22] |
| K_4_Sm_2_[PS_4_]_2_[P_2_S6] | *C*2 | [SmS_8_] | [PS_4_] | [23] |
| KErP_2_S_7_ | *P*2_1_/*c* | [ErS_8_] | [PS_4_] | [24] |
| LiEuPSe_4_ | *Ama*2 | [EuSe_8_] | [PSe_4_] | [9] |
| Rb_3_CeP_2_Se_8_ | *P*2_1_/*c* | [CeSe_8_] | [PSe_4_] | [25] |
| Cs_3_GdP_2_Se_8_ | *P*2_1_/*c* | [GdSe_8_] | [PSe_4_] | [25] |
| Rb_2_CeP_2_Se_7_ | *P*2_1_/*n* | [CeSe_8_] | [PSe_4_] [P_2_Se_6_] | [25] |
| Rb_2_GdP_2_Se_7_ | *P*2_1_/*n* | [GdSe_8_] | [PSe_4_] [P_2_Se_6_] | [25] |
| CsScP_2_S_7_ | *C*2 | [ScS_6_] | [P_2_S_7_] | This work |


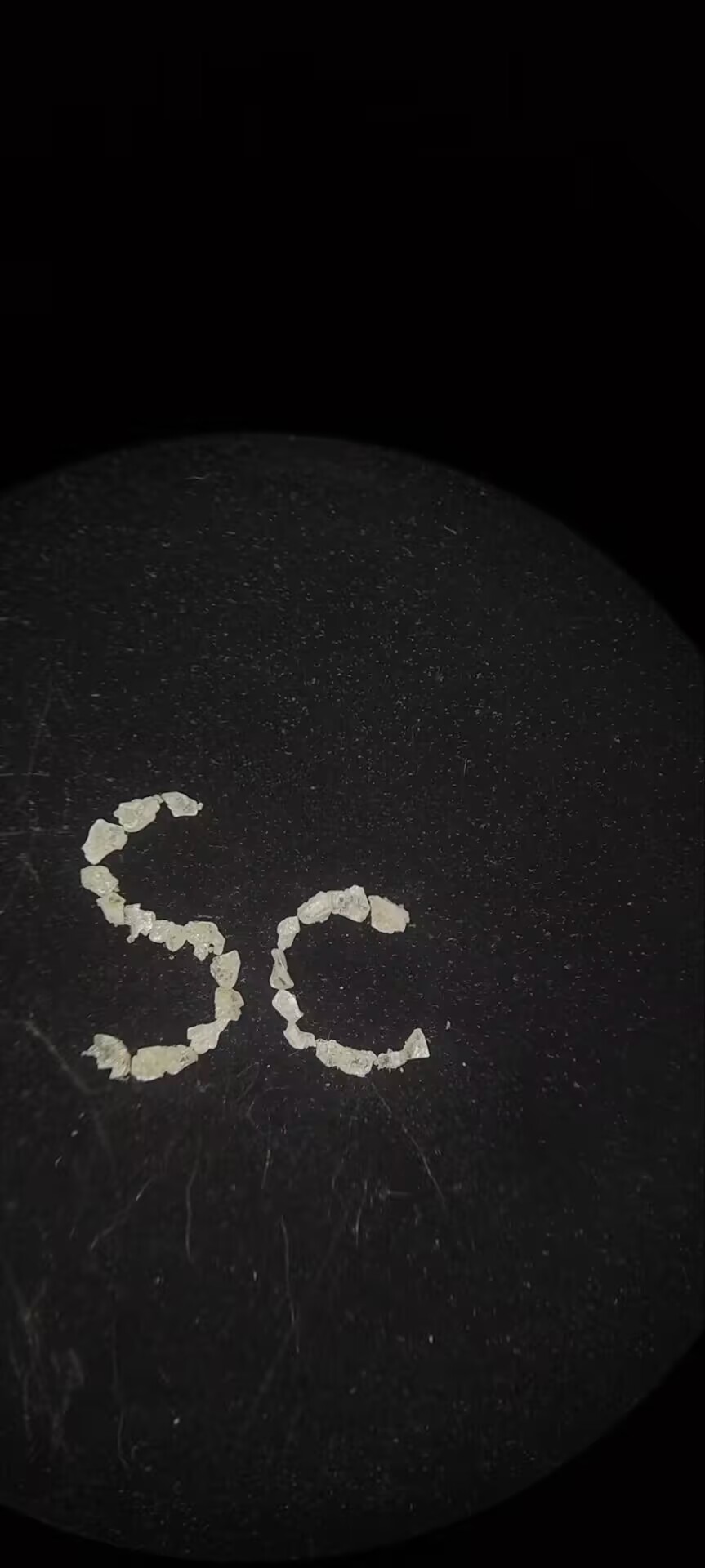


**Figure S1.** The crystal photograph of CsScP_2_S_7_.





**Figure S2.** The power X-ray diffraction pattern of CsScP_2_S_7_.





**Figure S3.** Band structure of CsScP_2_S_7_, and the Fermi level is set at 0 eV.





**Figure S4.** The calculated band structure of CsScP_2_S_7_ using HSE06.





**Figure S5.** Calculated frequency-dependent birefringence of CsScP_2_S_7_.





**Figure S6.** Calculated refractive indices of the fundamental and second harmonic wavelengths of CsScP_2_S_7_.

**Reference**

[1] X. Huang, S.-H. Yang, X.-H. Li, W. Liu, S.-P. Guo, *Angew. Chem. Int. Ed.* **2022**, *61* (32), e202206791.

[2] X. Tian, Y. Xiao, B. Zhang, D. Yang, K. Wu, *Mater. Today Phys.* **2022**, *28*, 100885.

[3] C.-Y. Zhao, W.-D. Yao, M.-Y. Li, W. Zhou, W. Liu, S.-P. Guo, *Inorg. Chem.* **2024**, *63* (9), 4017.

[4] Z.-X. Chen, C.-Y. Zhao, X.-H. Li, W.-D. Yao, W. Liu, S.-P. Guo, *Small* **2023**, *19* (8), 2206910.

[5] S. Milot, Y. Wu, C. Näther, W. Bensch, K. O. Klepp, *Z. Anorg. Allg. Chem.* **2008**, *634* (9), 1575.

[6] V. V. Klepov, K. A. Pace, L. S. Breton, V. Kocevski, T. M. Besmann, H.-C. zur Loye, *Inorg. Chem.* **2020**, *59* (3), 1905.

[7] X. Tian, Y. Xiao, B. Zhang, D. Yang, K. Wu, *Materials Today Physics* **2022**, *28*.

[8] Evenson, P. K. Dorhout, *Inorg. Chem.* **2001**, *40* (12), 2884.

[9] J. A. Aitken, K. Chondroudis, V. G. Young, M. G. Kanatzidis, *Inorg. Chem.* **2000**, *39* (7), 1525.

[10] T. Komm, S. Strobel, T. Schleid, *J. Alloys Compd.* **2008**, *451* (1-2), 648.

[11] Y. Wu, W. Bensch, *Inorg. Chem.* **2008**, *47* (17), 7523.

[12] P. L. Lange, S. Bette, S. Strobel, R. E. Dinnebier, T. Schleid, *Crystals* **2023**, *13* (10).

[13] L. S. Breton, M. D. Smith, H.-C. zur Loye, *CrystEngComm* **2021**, *23* (30), 5241.

[14] P. L. Lange, T. Schleid, *Eur. J. Inorg. Chem.* **2021**, *2021* (32), 3247.

[15] T. Komm, T. Schleid, *Z. Anorg. Allg. Chem.* **2004**, *630* (5), 712.

[16] P. L. Lange, R. Merkle, J. Maier, T. Schleid, *Solid State Sci.* **2023**, *143*.

[17] J. A. Aitken, M. G. Kanatzidis, *J. Am. Chem. Soc.* **2004**, *126* (38), 11780.

[18] T. Komm, T. Schleid, *J. Alloys Compd.* **2006**, *418* (1-2), 106.

[19] P. L. Lange, T. Komm, T. Schleid, *Z. Anorg. Allg. Chem.* **2021**, *647* (22), 2113.

[20] P. L. Lange, T. Schleid, *Z. Naturforsch. B* **2021**, *76* (5), 281.

[21] C. Müller, S. Jörgens, A. Mewis, *Z. Anorg. Allg. Chem.* **2007**, *633* (10), 1633.

[22] T. Schleid, I. Hartenbach, T. Komm, *Z. Anorg. Allg. Chem.* **2002**, *628* (1), 7.

[23] V. Manríquez, A. Galdámez, D. Guzmán-Águila, *Mater. Res. Bull.* **2008**, *43* (8), 2469.

[24] T. Komm, T. Schleid, *Z. Anorg. Allg. Chem.* **2005**, *632* (1), 42.

[25] K. Chondroudis, M. G. Kanatzidis, *Inorg. Chem.* **1998**, *37* (15), 3792.
